# Tendon Stem/Progenitor Cell Subpopulations and Their Implications in Tendon Biology

**DOI:** 10.3389/fcell.2021.631272

**Published:** 2021-02-18

**Authors:** Zizhan Huang, Zi Yin, Jialu Xu, Yang Fei, Boon Chin Heng, Xuesheng Jiang, Weishan Chen, Weiliang Shen

**Affiliations:** ^1^Department of Orthopedic Surgery, The Second Affiliated Hospital, School of Medicine, Zhejiang University, Hangzhou, China; ^2^Orthopedics Research Institute, Zhejiang University, Hangzhou, China; ^3^Institute of Sports Medicine, Zhejiang University, Hangzhou, China; ^4^Dr. Li Dak Sum and Yip Yio Chin Center for Stem Cell and Regenerative Medicine, Zhejiang University, Hangzhou, China; ^5^China Orthopedic Regenerative Medicine (CORMed), Hangzhou, China; ^6^Department of Infectious Diseases, The First Affiliated Hospital, Wenzhou Medical University, Wenzhou, China; ^7^School of Stomatology, Peking University, Beijing, China; ^8^Department of Orthopedic Surgery, Huzhou Hospital, Zhejiang University, Huzhou, China

**Keywords:** tendon stem/progenitor cells, subpopulation, niche, healing, TGFβ

## Abstract

Tendon harbors a cell population that possesses stem cell characteristics such as clonogenicity, multipotency and self-renewal capacity, commonly referred to as tendon stem/progenitor cells (TSPCs). Various techniques have been employed to study how TSPCs are implicated in tendon development, homeostasis and healing. Recent advances in single-cell analysis have enabled much progress in identifying and characterizing distinct subpopulations of TSPCs, which provides a more comprehensive view of TSPCs function in tendon biology. Understanding the mechanisms of physiological and pathological processes regulated by TSPCs, especially a particular subpopulation, would greatly benefit treatment of diseased tendons. Here, we summarize the current scientific literature on the various subpopulations of TSPCs, and discuss how TSPCs can contribute to tissue homeostasis and pathogenesis, as well as examine the key modulatory signaling pathways that determine stem/progenitor cell state. A better understanding of the roles that TSPCs play in tendon biology may facilitate the development of novel treatment strategies for tendon diseases.

## Introduction

**FIGURE 1:**
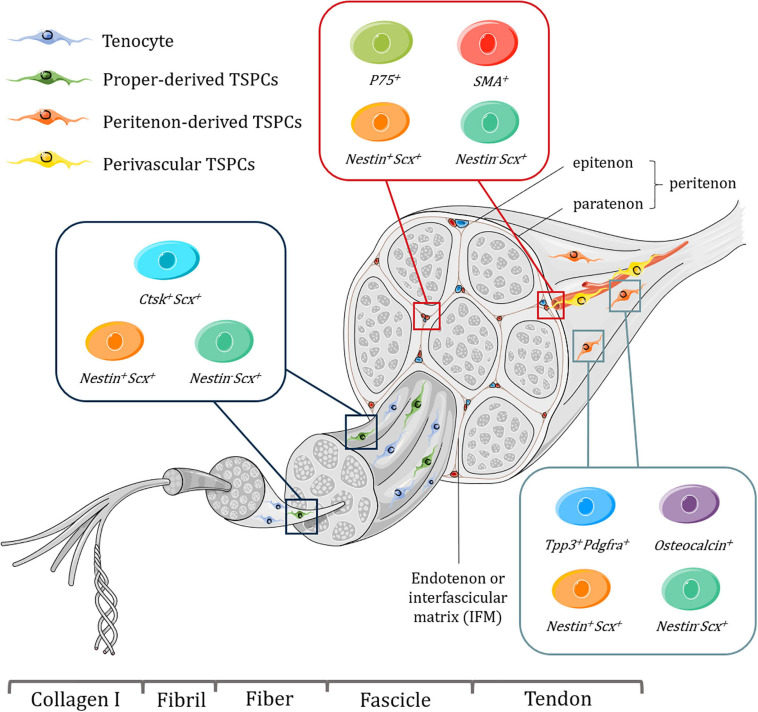
Schematic representation of tendon hierarchical structure and various subpopulations of TSPCs with specific markers harvested from different niches, including tendon proper, peritenon and perivascular region. Tenocytes are aligned between fibers. It should be noted that some of these subpopulations might overlap with each other and perivascular TSPCs may be present in endotenon as well as the peritenon. What’s more, the exact location of proper-derived TSPCs is not well determined. Figures were produced using Servier Medical Art (https://smart.servier.com/).

Tendon tissues have a hierarchical structure with unique mechanical properties, and serve to connect embryologically distinct musculoskeletal tissues, bone and muscle, and mainly function to transmit mechanical forces to enable skeletal locomotion. Tendons consist of fibrillar arrangement where type I collagen form fibrils, fibrils assemble into fibers, and then fibers assemble into fascicles (Nourissat et al., 2015). Bundles of fascicles form the fascicular matrix (FM) (Zhang et al., 2019). Endotenon or interfascicular matrix (IFM), a connective tissue compartment envelops each fascicle and is encompassed by the epitenon, which is covered by another layer of connective tissue, paratenon (Zhang et al., 2019). Together, the epitenon and paratenon are called peritenon (Mienaltowski et al., 2014). Tendon proper, refers to the remaining tendon tissue that comprises both FM and IFM after removing the peritenon (Mienaltowski et al., 2013; Zhang et al., 2019; Figure 1).

Tendon injuries remains a formidable challenge in the clinic, as disrupted tendon structure compromises tendon function and may lead to flawed healing, such as heterotopic ossification (HO) (Agarwal et al., 2016; Millar et al., 2017). Although surgical treatment can restore tendon tissue integrity, the injured tendon often cannot revert back to pre-injury conditions because of scar formation and fibrosis, which leads to higher risks of re-rupture (Andarawis-Puri et al., 2015). Multiple therapeutic modalities have been proposed to treat the disease such as platelet-rich plasma, hyaluronic acid, corticosteroid injection and so on (Osti et al., 2015; Frizziero et al., 2019; Kaux et al., 2019). Amongst these, stem cell-based treatment shows great promise, and tendon-derived stem cells (TDSCs) have aroused much interest due to their origin (Schneider et al., 2018).

Tendon stem cells (TSCs), commonly referred to as tendon stem/progenitor cells (TSPCs) due to their heterogeneity, exhibit varying propensities in differentiation potential. When these cells were first discovered, they were defined by their clonogenicity, self-renewal potential and multipotency (Bi et al., 2007). Since then, TSPCs have attracted a lot of attention because current treatment modalities for tendon diseases often fail to yield a satisfactory outcome. TSPCs play key roles in tendon development, homeostasis and healing (Bi et al., 2007). Transplantation of exogenous stem cells or activation of the endogenous population has already shown pro-regenerative effects on injured or diseased tendons (Schneider et al., 2018). Investigating the role of TSPCs in tendon biology is critical for unveiling the peculiar characteristics of tendon tissues. Better understanding and in-depth analysis of their identities, interaction with the local niche and involvement in the reparative process could promote optimized manipulation of TSPCs and hasten progress of future clinical applications.

Advancement in high-throughput sequencing and lineage tracing has made isolation and identification of distinct tendon stem cell subpopulations tangible, which further reveals distinct properties of TSPCs (Yin et al., 2016; Harvey et al., 2019). Other state-of-the-art technologies including genetic models and three-dimensional imaging, provide a means of dissecting the role of TSPCs in physiological and pathological processes of tendon tissues. Moreover, accumulating scientific evidence support the key roles of the TGFβ superfamily in determining the lineage fate of TSPCs (Tan et al., 2020).

This review will primarily focus on: (1) different subpopulations of TSPCs, (2) an overview of how TSPCs maintain tendon integrity, (3) the role of the TGFβ superfamily in regulating TSPCs lineage fate. We would like to address the latest discoveries of the emerging roles of TSPCs in tendon biology and pathology. Under most circumstances, TSCs, tendon progenitor cells (TPCs) or TDSCs should be included within the generic term of TSPCs.

## Subpopulations of TSPCs

### Niches of TSPCs

Stem cell niches dynamically orchestrate cell behavior and cell fate thorough physical interaction and regulatory factors. The native environment is critical for maintaining the stemness of TSPCs due to their topography and biological properties (Ning et al., 2015). Inherent topographical patterns, biochemical composition and biomechanical properties of native tendon matrix could facilitate homogeneous distribution and alignment, promote proliferation, and favor tenogenic phenotype instead of non-tenogenic differentiation of TSPCs (Yin et al., 2013; Ning et al., 2015).

Biglycan (Bgn) and fibromodulin, two critical extracellular matrix (ECM) components, have been shown to be crucial in regulating the lineage fate of TSPCs, since their depletion in double knock-out animal led instead to bone-like tissues being formed (Bi et al., 2007). Biglycan also enhances proliferation and tenogenic differentiation of TSPCs (Bi et al., 2007). Tenomodulin (Tnmd), a transmembrane glycoprotein with cleavable C-terminus localized on the ECM, is essential for adhesion to collagen I, and maintaining the self-renewal capacity, cell senescence and matrix remodeling capacity of TSPCs (Dex et al., 2017; Yin et al., 2019). But TSPCs still possess the multipotency after loss of tenomodulin (Alberton et al., 2015). Decellularized tendon matrix are superior in promoting proliferation and preserving stemness of TSPCs than other engineered biomaterial, which confirms the supportive role of tendon ECM in TSPCs maintenance (Zhang et al., 2011). Tendon ECM also favors the tenogenic disposition of TSPCs, which could be attributed to the niche signals of the tendon matrix (Yin et al., 2013). Alteration to ECM composition is frequently observed in tendinopathy, and aberrant differentiation of TSPCs could be induced by inflammatory and biomechanical cues, which accounts for the regulatory roles of local niches and their functions in tendinopathy (Xu and Murrell, 2008; Zhang and Wang, 2010c). The cellular component is critical in constituting the microenvironment as well, since non-stem/progenitor cells could secret paracrine factors to regulate the differentiation of TSPCs (Lee et al., 2015).

Perivascular regions have often been proposed as a potential niche for TSPCs. An early study had found that cells in the perivascular niche express stem cell-like characteristics (Tempfer et al., 2009). In fact, predominantly perivascular CD146-positive cells have been identified to constitute a fraction of the whole stem/progenitor population (Lee et al., 2015). CD146 is a commonly used marker to identify pericyte population (Gumucio et al., 2020). By utilizing Monocle pseudotime analysis, recent research has confirmed that pericytes form a part of the TSPCs population (De Micheli et al., 2020). Moreover, Xu et al., have reported a P75 (p75 neurotrophin receptor) expressing cell subpopulation with stem cell characteristics within the perivascular regions which could proliferate within the peritenon and migrate to interstitial space in response to injury (Xu et al., 2015). Finally, single-cell surface proteomics identified a perivascular niche where a tendon cell cluster expressed high levels of CD90 and CD146 (Kendal et al., 2020). Taken together, these results support the perivascular areas as a tendon stem cell niche.

In general, tendon stem cell niche is essential for TSPCs to maintain their properties and determine tenogenic fate.

### Early Insight of the Presence of Subpopulations Within the TSPCs Niche

Initial study had observed that TSPCs isolated from tendon proper actually consist of various phenotypes with heterogenous proliferation and differentiation capacities (Bi et al., 2007). The TSPCs’ lines of quadra-potential cells (i.e., tenogenesis, chondrogenesis, osteogenesis, and adipogenesis), yielded the highest expression levels of Scleraxis (Scx) and Mohawk (Mkx), probably suggesting their optimal tenogenic lineage commitment (Rajpar and Barrett, 2020). TSPCs derived from different anatomical origins at different developmental stages also exhibited distinct response to bioactive molecules, suggesting the heterogenicity of TSPCs (Brown et al., 2014). In addition, TSPCs extracted from discrete locations, in both the endotenon and peritenon, have different capabilities to form tendon-like construct (Mienaltowski et al., 2014).

Traditionally, TSPCs have been isolated from the tendon proper (Bi et al., 2007; Rui et al., 2010). However, cell population from the peritenon has been demonstrated to be capable of multipotent differentiation and migration (Cadby et al., 2014). They expressed higher amounts of progenitor cell markers including CD45, CD90, CD105, and Oct-4, and despite their relatively lower proportions, peritenon-derived stem/progenitor cells have higher proliferative capacity (Mienaltowski et al., 2013; Cadby et al., 2014). Upon labeling stem cells with Iododeoxyuridine (IdU), more label-retaining stem cells were found in the peritenon than in mid-substances, particularly at the perivascular region (Tan et al., 2013). Cells from the peritenon would activate Scleraxis expression in response to mechanical loading and could form primitive tendons *in vitro* (Mendias et al., 2012; Mienaltowski et al., 2013). Furthermore, following tendon injury, cells from the surrounding peritenon would proliferate, migrate and contribute to tenogenesis (Dyment et al., 2013; Tan et al., 2013; Sakabe et al., 2018). These phenomena are a reminder that TSPCs constitute heterogenous groups of cells with distinct characteristics.

### Recent Identification of Subpopulations Within the TSPCs Niche

The confirmation and lineage mapping of distinct TSPC subpopulations have been achieved by lineage tracing and single-cell sequencing (Figure 1). Early attempts at understanding the origin and identity of resident tendon progenitors mainly depend on lineage-tracing and alpha smooth muscle actin (αSMA) labeling revealed that SMA^+^ Scx^+^ cells located within the tendon mid-substance as an amplifying resident progenitor population that contribute to postnatal growth and healing (Dyment et al., 2014). Thus SMA^+^ cells could be a source of TSPCs. In a later study, Harvey et al. (2019) proposed that SMA^+^ cells are not TSPCs, as they did not convert to tenocytes with longitudinally aligned collagen matrix with second harmonic generation signals. However, a significant fraction of the SMA^+^ population did turn on Scx expression, which is a hallmark for tenogenesis (Dyment et al., 2014).

During the reparative process, diverse subpopulations of TSPCs could also be noticed, as TSPCs integrated into the injury site and they mainly constituted two subpopulations, with or without surface marker CD105 (Asai et al., 2014). The CD105-positive subpopulation perform better with regard to expressing Scx and avoiding chondroid degenerative lesions than the CD105-negative subpopulation (Asai et al., 2014). However, their specific origin is unclear.

Since then, intense efforts have been made to characterize subtleties within the tendon stem cell population. Our understanding of subpopulations of resident TSPCs has been improved greatly as potent single-cell sequencing method emerges and render a panoramic view of their composition. An important finding is that a nestin^+^ subpopulation of TSPCs, which is more capable of self-renewal and tenogenic differentiation than the nestin^–^ subpopulation, has been identified by single-cell analysis and is involved in the development and endogenous repair of tendon tissues (Yin et al., 2016). The majority of the nestin^+^ subpopulation reside in the endotenon and peritenon, particularly within the perivascular area (Yin et al., 2016). Additionally, nestin has been shown to be essential for maintaining the tenocyte-lineage phenotype and reparative capacities of TSPCs (Yin et al., 2016).

Transcriptome profiles revealed that peritenon harbors a collection of cell population and might be an abundant source of TSPCs (Mienaltowski et al., 2019). Indeed, Osteocalcin-expressing cells whose proliferation and differentiation are regulated by Hedgehog (Hh) signaling, have been found in the peritenon, demonstrating stem/progenitor cell properties comparable to TSPCs isolated from the mid-substance (Wang et al., 2017).

Tubulin polymerization-promoting protein family member 3 (Tppp3) is the first discovered molecular marker that is expressed in the developing epitenon and paratenon (Staverosky et al., 2009). Recently, a paratenon-derived cell cluster expressing both Tppp3 and platelet-derived growth factor receptor alpha (Pdgfra) has been identified as a novel subpopulation of tendon stem cells by utilizing single-cell transcriptomics, which are capable of self-renewal and generating *de novo* tenocytes (Harvey et al., 2019). Tppp3+Pdgfra+ cells dwell in the tendon sheath and are present from embryo to adulthood (Harvey et al., 2019). Unlike previously described TSPCs, the Tppp3+Pdgfra+ subpopulation express high levels of CD34 and rarely Scx (Harvey et al., 2019).

Tendon sheathes normally envelop areas of tendon fibers subjected to high levels of friction, and are conventionally believed to function as lubrication during movement. The pool and regenerative potential of tendon sheath stem/progenitor cells could add extra protection for vulnerable tendon.

The proper-derived and peritenon-derived stem/progenitor cells showed some differences. Proper-derived progenitors have greater potential in forming tendon-like structures compared to peritenon-derived progenitors (Mienaltowski et al., 2014). The peritenon-derived population has also been shown to secrete stimulatory factors that regulate tendon-related gene expression, such as Scx, Tnmd and Bgn (Mienaltowski et al., 2014). Furthermore, tendon proper-derived stem cells expressed genes related to cartilage and chondrocyte development, while peritenon-derived stem cells expressed genes related to positive regulation of endothelial cell proliferation and angiogenesis (Mienaltowski et al., 2019).

Recently, a report noted that the rat model to study tendon biology possess a different hierarchical structure compared to larger species, which lacks the structure of fascicle and hence the structure of endotenon (Lee and Elliott, 2019). Considering this, the TSPCs niche found in murine model might be different from that of larger species and future researchers should be cautious about animal model choice when they attempt to locate TSPCs niche in a more detailed scale.

### Potential New Source of TSPCs Subpopulations

A previous study has shown some evidence that adjoining tissues might provide a pool of stem/progenitor cells for tendon maintenance. The expanded SMA^+^ cells with negative Scx expression were initially present within surrounding structures (i.e., retinaculum and periosteum), then they migrated to the paratenon and later differentiated into the tenogenic lineage in response to injury, which indicates that adjacent paratendinous structures may serve as reservoirs of TSPCs (Dyment et al., 2014).

Recently, a research based on the zebra fish model demonstrated that progenitors from neighboring tissues are able to regenerate well-organized tendons after total ablation of embryonic tendon cells (Niu et al., 2020). At the surrounding cartilage or muscle attachment site, sox10^+^ perichondral cells and nkx2.5^+^ cells could generate a pool of progenitors capable of coordinating tendon regeneration (Niu et al., 2020). Since zebrafish tendons are structurally, molecularly and mechanically similar to mammalian tendons, the regenerative mechanism might shed light on the potential existence of adjoining tissues-derived stem cell subpopulations. Indeed, a recent study has defined an interstitial Scx^+^ cell subpopulations capable of tenogenic differentiation in adult skeletal muscles by single-cell analysis, which suggests a potential reservoir for tendon regeneration (Giordani et al., 2019). Furthermore, Hic1 successfully defined a subpopulation of vasculature-related Scx positive cells expressing Col22a1 within the peritenon near the myotendinous junction (MTJ), and they share unique but overlapping transcriptional properties with that of tendon progenitors (Scott et al., 2019). This very subpopulation is found to expand and are present within the tendon after muscle injury (Scott et al., 2019).

In tendon tissues, clusters of *ITGA7*^+^ cells, which are highly similar to those smooth muscle-mesenchymal cells found in muscle are situated around vessels and they also express surface markers CD90 and CD146 (Giordani et al., 2019; Kendal et al., 2020). Actually, developmental evidence suggests that TGFβ signaling emanating from muscles and cartilage are critical for tendon progenitors recruitment, implicating the cross-talk between different tissues of the musculoskeletal system (Pryce et al., 2009). Scx^+^Sox9^+^ progenitors give rise to the junction between the cartilage and tendon (Blitz et al., 2013; Sugimoto et al., 2013). The aforementioned evidences corroborate that musculoskeletal tissues might contain respective stem/progenitor populations. Future studies could investigate whether bone or muscle tissues from human or mouse contain a reserve cell population that share something in common with TSPCs, and which could restore functional tendon after injury. These efforts may provide novel cell sources for developing cell-based treatment.

### The Need for Novel Biomarkers to Trace TSPCs Subpopulations

Scx alone labels most but not all tendon cells (Sakabe et al., 2018). Besides Scx, tendon cells also express S100a4 which may help mark subsets of resident tendon stem cells, as S100a4 combined with Scx, label distinct but overlapping tendon cell subpopulations during homeostasis and healing (Best and Loiselle, 2019). Recent single-cell sequencing and Cellular Indexing of Transcriptomes and Epitopes by Sequencing (CITE-seq) results unveiled other previously unidentified tendon cell populations (De Micheli et al., 2020; Kendal et al., 2020). Canonical tenogenic markers Scx, Mkx and tenomodulin were only observed to be expressed in a subset of tenocytes and not necessarily co-expressed, which suggests great heterogenicity in tendon cells with different origins or functions (De Micheli et al., 2020). Characterization and classification of TSPCs by reliable and definitive markers are strongly needed to further map their distinct subpopulations and biological functions in tendon, because current markers including Oct-4, Nanog, Sox2, CD44 and Sca-1 are not very specific for labeling TSPCs (Tan et al., 2013; Table 1). Specific surface markers are also required to better isolate, sort and purify TSPCs, and thus to achieve better clinical applications.

**TABLE 1:** Niches and Markers of TSPCs.

**Species**	**Anatomical location**	**Niches**	**Markers**	**References**
Human	Hamstring tendon	Not determined	Tnmd, Stro1, CD146, CD44, CD90	Bi et al.,2007
Human	Hamstring tendon	Not determined	CD44, CD146, Stro1, αSMA, Tnmd	Ruzzini et al.,2014
Human	Supraspinatus tendon	Perivascular region	Musashi1, Nestin, Scx, SMA, Prominin1/CD133, Col I, Col III, Smad8, CD29, CD44	Tempfer et al.,2009
Human	Supraspinatus tendon	Not determined	CD90, CD105, CD73	Menon et al.,2018
Human	Patellar tendon	Tendon proper	CD44, CD73, CD90, CD105	Lee et al.,2012
Human	Achilles tendon	Not determined	CD105, CD90, CD44, CD146	Yin et al.,2010
Human	Achilles tendon	Not determined	CD73, CD90, CD105, Stro1, CD146, CD44, Musashi1	Kohler et al.,2013
Human	Achilles tendon	Not determined	Nestin, Scx, CD146, CD44, CD90	Yin et al.,2016
Human	Achilles tendon	Not determined	CD44, CD90	Hu et al.,2017
Human	Achilles tendon	Not determined	CD44, CD29, CD105, CD90	Qin et al.,2020
Mouse	Patellar tendon	Tendon proper	Comp, Scx, Sca1, Tenascin C, Col I, CD90.2, CD44, Sox9, Runx2	Bi et al.,2007
Mouse	Patellar tendon	Peritenon	Tppp3, Pdgfra, CD34	Harvey et al.,2019
Mouse	Achilles tendon	Tendon proper	Sca1, CD90.2, CD44, Tnmd, Scx, nucleostemin	Mienaltowski et al.,2013
Mouse	Achilles tendon	Peritenon	Sca1, CD90.2, CD44, endomucin, CD133, nucleostemin, Musashi1	Mienaltowski et al.,2013
Mouse	Achilles tendon	Not determined	CD29, CD44, CD49e, Sca1	Asai et al.,2014
Mouse	Achilles tendon	Tendon proper	Nestin, Scx, CD146, CD105, CD90, CD44, CD29, CD51	Yin et al.,2016
Mouse	Achilles tendon	Tendon proper	Fmod, Mkx, Gdf5, Scx, Thbs4, Wnt10a	Mienaltowski et al.,2019
Mouse	Achilles tendon	Peritenon	Prominin1/CD133	Mienaltowski et al.,2019
Mouse	Achilles tendon	Tendon proper	Ctsk, Nestin, Sca-1, CD44, CD105, CD24, CD200	Feng et al.,2020
Mouse	Tail tendon	Not determined	CD146, CD105, CD90.2, CD73, CD44, Sca1, Nestin, Nanog	Alberton et al.,2015
Mouse	Tail tendon	Not determined	CD90.2, Sca1	Liu et al.,2017
Mouse	Tibialis Anterior Tendon	Peritenon	Osteocalcin	Wang et al.,2017
Mouse	Limb tendon	Peritenon	Sca1, CD34, CD44	Tan et al.,2020
Rat	Flexor tendon	Tendon proper	CD44, CD90, Tenascin C, Tnmd, Aggrecan, αSMA	Rui et al.,2010
Rat	Patellar tendon	Tendon proper	Nucleostemin, Scx, Tnmd, Oct4, SSEA4, CD44, CD90.1	Zhou et al.,2010
Rat	Patellar tendon	Tendon proper	CD73, CD90, Scx, Tnmd	Tan et al.,2012
Rat	Patellar tendon	Not determined	CD146, CD44, Sca1, Scx, Tnmd, Smad8, Oct4, Nanog, Sox2, nucleostemin	Tan et al.,2013
Rat	Patellar Tendon	Perivascular region	CD29, CD90, P75, Vimentin, Sox10, Snail	Xu et al.,2015
Rat	Achilles tendon	Not determined	Nucleostemin, Oct 3/4, Dyn2	Runesson et al.,2015
Rat	Achilles tendon	Not determined	CD29, CD44, CD90	Chen et al.,2016
Rat	Achilles tendon	Tendon proper	CD90, CD73, nucleostemin	Guo et al.,2016
Rat	Achilles tendon	Not determined	CD44, Stro1	Hu et al.,2016
Rabbit	Patellar tendon and Achilles tendon	Tendon proper	Oct4, SSEA4, nucleostemin	Zhang and Wang,2010a
Horse	Superficial digital flexor tendon	Tendon proper	Scx, CD90, CD105, Oct4	Cadby et al.,2014
Horse	Superficial digital flexor tendon	Peritenon	CD45, CD90, CD105, Oct4	Cadby et al.,2014
Horse	Superficial digital flexor tendon	Tendon proper	CD44, CD90, CD29	Durgam et al.,2019

Hierarchically-expressed markers that can reveal the origin and development of stem/progenitor cells might be uncovered. TSPCs exhibit different characteristics on the spatiotemporal scale, which might correlate to specific stages of development (Chen et al., 2016). Combined single-cell RNA sequencing with genetic-based lineage tracing, stemness markers and spatial information would enable us to better understand the various subpopulations, their characteristics and functions *in vivo* and sequential stem cell states. Elucidation of how different subpopulations contribute to regeneration and most importantly the role that they play in yielding non-functional scar formation and heterotopic ossification, might enable formulation of more targeted strategies to improve tendon healing.

It must however be noted that viable markers for the identification of TSPCs *in vitro* are not necessarily useful for tracking TSPCs *in situ*. Tendon tissues across the body and between different species differ in architecture, biomechanics and transcriptome (Disser et al., 2020). Much caution should be exercised in classifying TSPCs subpopulations, considering the influence of different cell sources and contamination. More specific markers would allow precise fate-mapping of ambiguous stem/progenitor populations. A more specific culture system should be developed, as the traditional culture system fails to maintain the phenotype of TSPCs (Yan et al., 2018; Zhang et al., 2018).

## TSPCs in Tendon Biology

### TSPCs Participate in Tendon Homeostasis

Tendon maintenance involves not only its extracellular matrix, but also the cells that reside within it. The cell-ECM interaction is essential for maintaining tendon homeostasis as ECM could generate cell signals that regulate proliferation, differentiation, adhesion and migration (Screen et al., 2015). Although TSPCs were previously thought to be dormant in healthy adult tendon without injury, the shifted postnatal cell turnover activity of tendon unveiled the possibility of resident tendon stem/progenitor population participating in the homeostatic renewal mechanism (Runesson et al., 2013; Grinstein et al., 2019). A recent study showed a transitional cell division rate and dynamic tendon-related gene expression in postnatal tendon tissues (Grinstein et al., 2019). In fact, TSPCs are capable of adjusting gene expression and modifying ECM in response to different mechanical loadings, which favors the expression of tenocyte-related genes at moderate levels (Zhang and Wang, 2010b). The *in vivo* roles of TSPCs within intact tendon remain largely elusive since most studies investigating TSPCs activity are conducted in the context of injury or ex vivo models. Further exploration into their *in vivo* activities is needed.

### TSPCs Plays an Essential Role in Tendon Regeneration

The origin or source of cells that contribute to the tendon healing process have not been fully elucidated. TSPCs from both the tendon proper and surrounding peritenon are known to participate in the process of tendon repair, representing the intrinsic and extrinsic response to tendon injuries (Bi et al., 2007; Harvey et al., 2019). Intrinsic recruitment of Scx^+^ cells are critical for restoring tendon, which accounts for the superior regeneration observed in neonates compared to adults, since adult Scx^+^ cells are not mobilized properly and transdifferentiate into ectopic cartilage (Howell et al., 2017). A nestin^+^Scx^+^ subpopulation would be recruited to the injury site within a short time period (Yin et al., 2016). TSPCs are supposed to differentiate into the tenogenic-lineage in response to tendon injury. As expected, at 14 days post-injury, Scx-positive lineage cells have been integrated into the aligned bridging tissues that connect two ends of transected sites (Best and Loiselle, 2019).

The paratenon transforms from a quiescent state to an active state, generating multiple cell layers and bridging the wound site and cells within it, which would turn on expression of tenogenic markers, such as Scx (Dyment et al., 2013). The cells from the periphery of the struts would also express Scx, which indicates a possible Scx-negative stem cell subpopulation (Dyment et al., 2013). Following injury, an expanded SMA^+^ population within paratenon would form a collagenous bridge and permeate nearby tendon struts where high level tenascin-C could be detected as they remodel the tendon body (Dyment et al., 2014). The collagen fibers of the bridge would transform from loose and thin to dense and thick as time progresses (Dyment et al., 2014). There are almost no Scx-positive lineage cells, which indicates the origin of tendon proper, exhibiting αSMA staining in normal tendon and bridging scar tissues (Howell et al., 2017; Best and Loiselle, 2019).

Furthermore, sheath osteocalcin-expressing stem/progenitor cells will congregate at the injury site, differentiate into tenocytes, and engender fiber-like structures, during which activated Hh signaling is critical for the reparative capacity of sheath stem/progenitor cells (Wang et al., 2017; Table 2).

**TABLE 2:** Behaviors of tendon cells during regeneration.

**Tendon cell populations**	**Events**	**References**
Paratenon cells	3 d.p.i. Cells proliferate and produce tenascin-C and fibromodulin 7 d.p.i. Migrate toward the lesion and express Scx and smooth muscle actin alpha, maintain tenascin-C and fibromodulin expression 14 d.p.i. Bridge the lesion	Dyment et al.,2013
SMA^+^ cells	7 d.p.i. Partly Migrate from adjacent structure, expanded in the paratenon and synthesize collagen in paratenon bridge 14 d.p.i. Extend over the lesion, infiltrate adjacent region to remodel and mostly differentiate into Scx^+^ cells; Bridge formed 35 d.p.i. Reduced SMA^+^Scx^+^ cells	Dyment et al.,2014
P75^+^ cells	0–7 d.p.i. Cells proliferate 2 d.p.i. Capillaries formed; contact with endothelial cells in the peg and socket arrangement; detached from basal lamina encasement; deposit ECM 28 d.p.i. Cell number decreases	Xu et al.,2015
Nestin^+^Scx^+^ cells	7 d.p.i. Accumulated at the injury site 7–21 d.p.i. Cell number decreased	Yin et al.,2016
Osteocalcin^+^ cells	14 d.p.i. Migrate to lesion; Express Mkx 45 d.p.i. Form tendon-fiber-like construct, Mkx. Scx, ECM components significantly upregulated	Wang et al.,2017
Tppp3^+^Pdgfra^+^ cells	3–14 d.p.i. Migrate to lesion 3–7 d.p.i. Turn on Scx 14 d.p.i. Located deep within mid-substance 1–14 d.p.i. Primarily proliferate, peak at 7 d.p.i., cease at 28 d.p.i.	Harvey et al.,2019
Scx^+^ lineage cells	0–2 d.p.i No Scx^+^. Present within the scar tissue 14 d.p.i. Present at the injury site 21 d.p.i. Specific to the tendon stubs and form aligned bridging region of the scar tissue	Sakabe et al., 2018; Best and Loiselle,2019

*d.p.i., days post injury.*

The majority of the Tppp3^+^ lineage inhabits the paratenon sheath and mostly remain quiescent in the homeostatic state (Harvey et al., 2019). Likewise, once tendon is injured, Tppp3^+^Pdgfra^+^ cells would migrate and infiltrate the mid-substance to repair the damaged region, where they differentiate into tenocytes and lose their stem cell signature (Harvey et al., 2019). Tppp3^+^Pdgfra^+^ stem cells left within sheath would proliferate and maintain their proportions (Harvey et al., 2019). During the healing process, PDGFRα signaling is indispensable for tenogenic differentiation of the Tppp3^+^Pdgfra^+^ subpopulation, but not necessary for their Scx expression (Harvey et al., 2019). Also, a small fraction of this subpopulation might contribute to fibrosis during tendon healing (Harvey et al., 2019). Inflammation is a vital part of the tendon healing process which could eventually impact the reparative outcome. TSPCs were demonstrated to play a regulatory role during inflammation and remodeling when encountering acute tendon injuries by upregulating IL-10 and TIMP-3 via the JNK/STAT signaling pathway (Tarafder et al., 2017). Inflammation could determine the fate of TSPCs as inflammatory signaling and mediators were shown to have effects on TSPCs (Hu et al., 2016). Abnormal upregulation of HIF-2α in proinflammatory milieu directs TSPCs commitment into osteochondral-lineage (Hu et al., 2016). Prostaglandin E_2_ decreases the proliferation capacity of TSPCs, and induces their non-tenogenic differentiation (Zhang and Wang, 2010c). IL-1β could promote the motility of TSPCs and also cause phenotype loss of TSPCs, which is associated with altered expression of tendon-related genes (Zhang et al., 2015; Chen et al., 2019; Wang et al., 2019b). Altogether, these evidences reveal a dynamic interplay between inflammation and TSPCs.

Notably, the proportions of TSPCs subpopulations vary according to tendon tissue types, which might determine the corresponding reparative outcome (Dyment et al., 2014; Harvey et al., 2019). Also, different populations display distinct regional and temporal expressions during the reparative process, whereby Oct3/4 positive cells are enriched at the injury site and nucleostemin positive cells are dispersed throughout tendon (Runesson et al., 2015).

It is also worth noting that embryonic and early postnatal tendons regenerate better than late postnatal tendons, which may be caused by the differences between embryonic and postnatal TSPCs (Beredjiklian et al., 2003; Howell et al., 2017). After inflammatory stimuli treatment, embryonic and postnatal tendon cells share similar tenogenic commitment but the latter upregulate expression of inflammatory mediators and catabolic enzymes (Li J. et al., 2019). TSPCs from embryo reside in a mechanically different tendon compared with TSPCs from postnatal stage and during development embryonic TSPCs become more tenogenesis guided (Nguyen et al., 2018). Additionally, mechanical loading alone hardly regulates the behavior of embryonic TSPCs while it can change the dynamic of postnatal TSPCs (Zhang et al., 2010; Brown et al., 2014).

**FIGURE 2:**
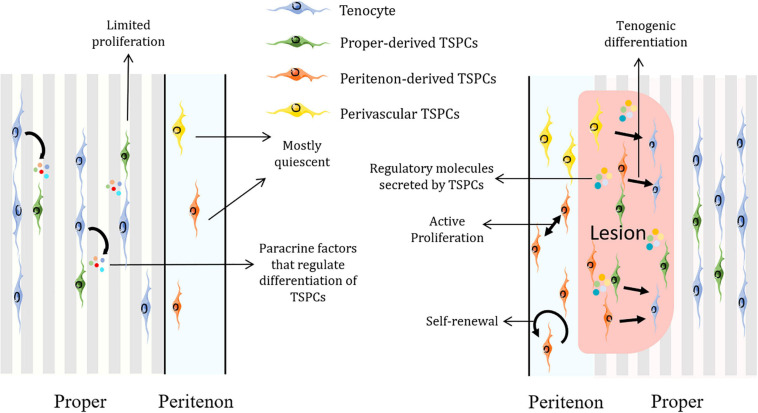
Schematic representation of how TSPCs are implicated in tendon homeostasis and regeneration. TSPCs remain mostly dormant in homeostatic state and surrounding non-stem/progenitor cells could secret factors to regulate their differentiation. Upon injury, TSPCs would be activated and mobilized. TSPCs self-renew, proliferate, migrate to the lesion and differentiate into tenocytes as well as modify the inflammatory process by increasing regulatory molecules such as IL-10. Figures were produced using Servier Medical Art (https://smart.servier.com/).

In general, TSPCs would migrate to the injury site, proliferate and express tendon-related, pluripotency and pericyte-related markers to modulate tendon healing and remodeling (Tan et al., 2013). Also, exosomes from TSPCs are capable of regulating matrix metabolism and promoting tenogenesis of TSPCs, which could further boost tendon healing (Wang et al., 2019a; Figure 2).

Accumulating scientific evidence shows that the treatment of TSPCs combined with proper delivery vehicle could promote tendon regeneration (Shen et al., 2012; Lui et al., 2014; Komatsu et al., 2016; Zhang et al., 2018). These validate the potential therapeutic efficacy of stem-cell based treatment. Investigating the mechanism that drives the switch from the quiescent to active state of TSPCs could benefit translational application. Spatiotemporal distribution and cellular dynamics of different subpopulations during the reparative process should be addressed, which will help us develop more precise manipulation of stem-cell based therapy to overcome the clinical barriers.

### TSPCs in Tendon Pathology

TSPCs from tendinopathic tissues exhibit altered characteristics, such as proliferation and differentiation capacity (Kim et al., 2018; Chang et al., 2020). The tendinopathic region showed strong expression of MKX and GLI1, protein product of the Hh target gene, indicating that Hh signaling is activated (Wang et al., 2017). The expression of higher collagen III to collagen I ratio is observed in a TSPCs phenotype, which is a pattern usually seen in tendinopathy (Rajpar and Barrett, 2020). Additionally, general factors related to tendinopathy also reshape TSPCs (Scott et al., 2015; Ranger et al., 2016). Hyperglycemia decreases the proliferation capacity of TSPCs as well as promotes their osteochondrogenic differentiation potential (Shi et al., 2019). High cholesterol not only inhibits tendon-related gene expressions in TSPCs but also initiates their apoptosis and autophagy (Li K. et al., 2019; Li et al., 2020).

Scx^+^ lineage progenitor cells have been recognized as the origin of ectopic bone formation (Dey et al., 2016; Agarwal et al., 2017). Rigorous studies on various subpopulations of TSPCs deepen our understanding of the elusive mechanism of tendon pathological healing. A Cathepsin K (Ctsk) expressing TSPCs subpopulation present at mid-substance had been identified, which contribute to heterotopic ossification (Feng et al., 2020). Ctsk is a proven marker of osteoclasts, periosteal stem cells and perichondrial progenitors (Nakamura et al., 2007; Yang et al., 2013; Debnath et al., 2018). Ctsk^+^Scx^+^ TSPCs possess great self-renewal capacity and differentiation potentials with enriched progenitor cell markers (Feng et al., 2020). The depletion of the Suppressor of fused followed by activation of Hh signaling would trigger subsequent chondrogenesis and osteogenesis of Ctsk^+^Scx^+^ TSPCs (Feng et al., 2020).

Aging has also been revealed as a contributor to the altered properties of TSPCs (Zhou et al., 2010). TSPCs isolated from aged or degenerated tendon exhibit early sign of senescence and shifted transcriptomic profiling with impaired self-renewal and clonogenic ability (Kohler et al., 2013; Ruzzini et al., 2014). Besides, aged TSPCs show reduced migratory capacity, slower actin turnover, and dysregulated gene expressions related to cell-matrix interactions (Kohler et al., 2013). These effects are at least partly caused by augmented Rho-associated protein kinases (ROCK), inhibition of which could restore the phenotype of aged TSPCs to one similar to young TSPCs (Kohler et al., 2013; Kiderlen et al., 2019). Additionally, downregulation of nuclear regulator CITED2 and Aquaporin 1(AQP1) are also reported to be correlated with TSPCs aging (Hu et al., 2017; Chen et al., 2020). Functionally incompetent aged TSPCs will assemble into a less cell-populated, poorly organized and biomechanically inferior three-dimensional tendon organoids (Yan et al., 2020). With advancing age, osteogenic differentiation potential and BMP expression are enhanced in TSPCs, which consequently contribute to increased heterotopic ossification in tendons (Dai et al., 2020). These researches confirm the profound effects of aging on the phenotype of TSPCs and explain the higher incidence of tendon disorders in elderly population (Gumucio et al., 2014).

Generally, TSPCs display shifted properties in tendon diseases and account for key processes in pathogenesis. How TSPCs transform and transdifferentiate under pathological conditions need to be addressed utilizing single-cell sequencing and lineage tracing, so as to identify diseases-specific TSPCs phenotype and develop more targeted therapeutic strategies.

### Major Roles of TGFβ Superfamily Signaling in TSPCs

The TGF superfamily include Transforming Growth Factor-beta (TGFβ), Bone Morphogenetic Proteins (BMPs), and Growth/differentiation Factor (GDF). The ligand-receptor model charting cell-interaction found that the TGFβ family and their ligands were among the most abundantly expressed growth factors within tendon tissues, indicating their significant roles in tendon biology (De Micheli et al., 2020).

TGFβ signaling enables tenocytes to retain a stable cellular phenotype (Theiss et al., 2015; Tan et al., 2020). TSPCs treated with TGFβ2 tend to undergo tenogenesis as TGFβ2 treatment increase both Col1a and Scx expression (Guerquin et al., 2013; Brown et al., 2014; Havis et al., 2014; Liu et al., 2015). It was reported that Mkx regulates tenogenesis by directly activating TGFβ2 in TSPCs (Liu et al., 2015). Additionally, transcription of EGR1 also directs tenogenic differentiation partially via TGFβ2 signaling (Guerquin et al., 2013). Endogenous microRNA MiR-378a could bind to TGFβ2, suppressing tenogenic differentiation of TSPCs and impeding tendon healing (Liu et al., 2019).

Transcriptome analysis reveals that during the developmental process of mouse limbs, TGFβ is the most predominant signaling pathway in tendon cells and shows the highest upregulation of TGFβ in differentiated tenocytes, as compared with TSPCs (Havis et al., 2014). TGFβ/SMAD2/3 signaling is required for Scx expression in undifferentiated tendon progenitors and essential for tendon development, which might regulate later recruitment of tendon cells (Pryce et al., 2009; Havis et al., 2016). Other than serving as a potent inducer of Scleraxis expression and a strong recruiter of tendon progenitors, TGFβ also maintained Scx expression of TSPCs *in vitro* (Asai et al., 2014; Feng et al., 2020). Additionally, TGFβ balance the expression of Sox9 and Scx expression within a pool of progenitor cells and thus modulate their chondrogenic and tenogenic differentiation (Blitz et al., 2013; Sugimoto et al., 2013).

After abrogation of TGFβ signaling, committed and functional tendon cells lost their differentiation markers and revert to a more stem/progenitor cell-like state, acquiring expression of stem cell markers such as Sca-1 and CD44 (Tan et al., 2020). Immediately after reintroduction of the TGFβ type II receptor, the mutant cells are able to recover differentiated fate (Tan et al., 2020). Nevertheless, a mere loss of TGFβ signaling alone is not sufficient to induce tendon cell dedifferentiation, since the microenvironment may also participate in the process (Tan et al., 2020). As is the case of early embryonic development, continuous TGFβ signaling with external factors is essential for tendon progenitors to undergo commitment to the tendon cell fate (Pryce et al., 2009). TGFβ would also be released in response to mechanical forces and thus modulate ECM production and sensory projections of tenocytes (Subramanian et al., 2018). All in all, TGFβ acts to maintain tenocyte morphology and differentiation fate (Subramanian et al., 2018; Tan et al., 2020).

CD105 has been marked to be a coreceptor of the TGFβ superfamily (Sakamoto et al., 2020). CD105-negative TSPCs collected from the injury site exhibited a stronger chondrogenic ability than the CD105-postive subpopulation, which are closely related to activated TGFβ/BMP signaling (Asai et al., 2014). Transforming Growth Factor B Induced Gene Human Clone 3 (βig-h3), an extracellular matrix protein induced by TGFβ, were found to be upregulated in Achilles tendon HO (Zhang et al., 2020). βig-h3 could bind to TSPCs and inhibit their attachment to collagen I, as well as accelerate the condensation of TSPCs and promote mesenchymal chondrogenesis (Zhang et al., 2020). Inhibition of TGFβ effectively attenuates HO progression at multiple stages (Wang et al., 2018). Amelioration of HO progression could also be achieved by inhibiting Hh signaling (Feng et al., 2020). However, Hh signaling promotes tendon healing by inducing Mkx and Collagen I expression of tendon sheath stem cell through the TGFβ pathway (Wang et al., 2017). These evidences show the complicated roles of TGFβ signaling in the context of TSPCs.

BMP signaling is critical to TSPCs. A complete ECM niche mediates TSPC fate by BMP signaling (Bi et al., 2007). The absence of biglycan and fibromodulin increased sensitivity of TSPCs to BMP2, and consequently promote osteochondrogenic differentiation (Bi et al., 2007). BMP2 could stimulate non-tenogenic differentiation of TSPCs and elevated BMPs are observed in tendinopathic samples (Rui et al., 2011, 2012, 2013). Scleraxis-lineage cells contribute to all stages of heterotopic ossification of tendon and hyperactive BMP receptor has been shown to be involved in the process of chondrogenesis (Dey et al., 2016; Agarwal et al., 2017). BMP signaling has also been shown to contribute to enthesis development in mouse embryos (Blitz et al., 2009). BMP signaling is active in zebrafish Sternohyoideus tendon formation and regeneration (Niu et al., 2020). Pharmacological or genetic inhibition of BMP signaling have been demonstrated to reduce the number of tendon cells and impede the attachment progenitor cells being recruited and becoming tendon cells (Niu et al., 2020).

GDF signaling exerts effects on TSPCs as well. Growth/differentiation factor 5 (GDF5) labeled progenitor population also participate in tendon formation as they extend from the tendon proper to enthesis (Dyment et al., 2014). GDF5 promotes the transition of TSPCs toward tenocytes (Holladay et al., 2016).

**FIGURE 3:**
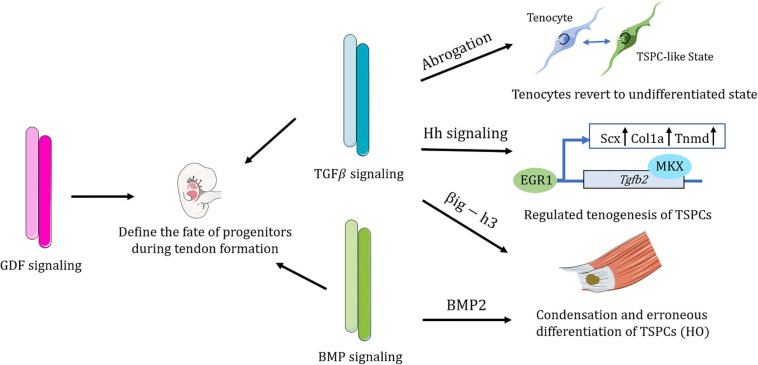
Schematic representation of the complex regulatory roles of the TGFβ superfamily in TSPCs. TGFβ signaling has been shown to maintain the differentiated fate of tenocytes, without which tenocytes would revert to a more TSPC-like state. TGFβ signaling directs distinct lineage commitment of TSPCs by different mechanisms. BMP signaling contributes to erroneous differentiation of TSPCs. TGFβ superfamily members, including TGFβ signaling, BMP signaling, and GDF signaling are all vital in defining the fate of tendon progenitors during tendon formation. Figures were produced using Servier Medical Art (https://smart.servier.com/).

In general, members of the TGFβ superfamily are essential molecular regulators of the orchestrated differentiation of tendon progenitors and tendon formation (Figure 3).

## Concluding Remarks and Future Directions

Tendon stem/progenitor cells are heterogenous and consist of several distinct but overlapping subpopulations. They are actively involved in tendon development, homeostasis and pathogenesis. Significantly, various members of the TGFβ superfamily play multiple roles in regulating TSPCs. Cutting-edge technology such as single-cell sequencing, multi-omics analyses and three-dimensional imaging, could provide researchers more potent tools to define and determine the dynamics and functions of the various TSPCs subpopulations. A full illustrative map would greatly benefit the development of more effective cell-based therapies and likely enable precision medicine.

Future studies could explore how TSPCs interact with neighboring cells, and the tissue environment to establish properly patterned tendon tissue and influence the regenerative process. Identifying other undiscovered TSPCs subpopulations and which of these is responsible for certain physiological or pathological process, are of great importance as well. Scientists could also elucidate more specific roles of the TGF-β superfamily and thus design corresponding strategies to address the clinical challenges brought by tendon rupture or tendinopathy.

## Author Contributions

ZH: original draft writing. ZY: conception and design. JX: visualization. YF: table preparation. BH, WC, and WS: review and editing draft. XJ: referenced manuscript analysis. All authors contributed to the article and approved the submitted version.

## Conflict of Interest

The authors declare that the research was conducted in the absence of any commercial or financial relationships that could be construed as a potential conflict of interest.
